# Haemodynamic Assessment and Outcomes of Aortic Valvuloplasty for Aortic Regurgitation in Patients with Bicuspid Aortic Valve

**DOI:** 10.3390/jcm13247544

**Published:** 2024-12-11

**Authors:** Kosuke Saku, Satoshi Arimura, Tomomitsu Takagi, Akihiro Masuzawa, Yoko Matsumura, Michio Yoshitake, Ryuichi Nagahori, Kenta Murotani, Takashi Kunihara

**Affiliations:** 1 Department of Cardiac Surgery, The Jikei University School of Medicine, 3-25-8 Nishishinbashi, Minato-ku, Tokyo 105-8461, Japan; satoshi.arimura@gmail.com (S.A.); tomomitsu-t@jikei.ac.jp (T.T.); amasuzaw-tky@umin.ac.jp (A.M.); yohkoh-m@jikei.ac.jp (Y.M.); myoshitake@jikei.ac.jp (M.Y.); nagahori@jikei.ac.jp (R.N.); kuniharat@gmail.com (T.K.); 2 Biostatistics Center, Kurume University School of Medicine, 67 Asahimachi, Kurume 830-0011, Japan; kmurotani@med.kurume-u.ac.jp; 3 Department of Cardiac Surgery, The Cardiovascular Institute, 3-2-19 Nishiazabu, Minato-ku, Tokyo 106-0031, Japan

**Keywords:** aortic valvuloplasty, aortic regurgitation, aortic valve replacement, bicuspid aortic valve, pressure gradient, reverse remodelling

## Abstract

**Background:** Aortic valvuloplasty for bicuspid aortic valve carries a risk of postoperative stenosis. We evaluated the haemodynamic differences between aortic valvuloplasty for bicuspid aortic valve, tricuspid aortic valve, and aortic valve replacement by echocardiography. We also assessed whether a higher postoperative pressure gradient affects the outcomes of aortic valvuloplasty for bicuspid aortic valve. **Methods:** From 2014 to 2021, patients undergoing aortic valvuloplasty were classified into aortic valvuloplasty for bicuspid aortic valve (Group-PB) and aortic valvuloplasty for tricuspid aortic valve (Group-PT). We also enrolled patients undergoing aortic valve replacement (Group-R) between 2002 and 2021. Mid-term outcomes were compared within Group-PB based on peak pressure gradients of ≥20 mmHg (subgroup-H) and <20 mmHg (subgroup-L). **Results:** Group-PB included 42 patients and Group-PT included 70 patients. Both 7-day and 1-year echocardiography showed the highest peak/mean pressure gradients in Group-PB (*n* = 41) and the lowest values in Group-PT (*n* = 67). Propensity scoring analysis yielded similar results to an unadjusted analysis. The mid-term outcomes were not significantly different between subgroup-H (*n* = 20) and subgroup-L (*n* = 22), with rates of freedom from aortic regurgitation >II at 5 years of 94.4% vs. 94.4% (*p* = 0.749) and freedom from reoperation of 94.4% vs. 100.0% (*p* = 0.317), respectively. **Conclusions:** Aortic valvuloplasty for tricuspid aortic valve shows favourable valve function in the early postoperative period, whereas aortic valvuloplasty for bicuspid aortic valve has a risk of postoperative stenosis. However, a high pressure gradient (peak pressure gradient of ≥20 mmHg) after aortic valvuloplasty for bicuspid aortic valve does not impact mid-term outcomes.

## 1. Introduction

Aortic valvuloplasty (AVP) has emerged as an alternative surgical option for aortic regurgitation (AR), and satisfactory long-term outcomes have been reported [1,2]. However, AVP for bicuspid aortic valve (BAV) is associated with a risk of postoperative stenosis [3]. On the other hand, aortic valve replacement (AVR) results in a smaller aortic valve area (AVA) than that before treatment, regardless of the type of prosthetic valve used [4]. We speculated that AVP for tricuspid aortic valve would show favourable valve function.

A high postoperative peak pressure gradient (PG), defined as ≥20 mmHg, negatively affects valve durability [5]. Therefore, it may be important to prevent residual postoperative PG. 

In the present study, we evaluated the haemodynamic differences between AVP for BAV, AVP for tricuspid aortic valve, and AVR. We also investigated the risk factors for residual relevant PG in patients with BAV. Furthermore, we compared the mid-term outcomes between a high-PG subgroup and low-PG subgroup after AVP for BAV.

## 2. Materials and Methods

### 2.1. Patients

Patients who underwent AVP at two institutions (the Jikei University School of Medicine and The Cardiovascular Institute) between March 2014 and October 2021 were divided into the following two groups: AVP for BAV (Group-PB) and AVP for tricuspid aortic valve (Group-PT). Patients who underwent valve-sparing aortic root replacement (VSRR) were included in this study as AVP. Patients undergoing emergent AVP for acute aortic dissection were excluded. Patients with unicuspid or quadricuspid aortic valve were also excluded. We also enrolled patients undergoing AVR for AR (Group-R) between November 2002 and October 2021. To compare the results of postoperative echocardiographic findings at 7 days and 1 year between each group, patients who underwent reoperation on the aortic valve within one year were excluded from the analysis. However, in the evaluation of postoperative outcomes in Group-PB, events that led to AVR through reoperation were not excluded from the analysis of mid-term outcomes.

### 2.2. Ethical Approval

The Jikei University School of Medicine and The Cardiovascular Institute approved the anonymous use of patient data in this retrospective study (ID:33-497 on 4 April 2022 and ID: 436 on 1 February 2023, respectively).

### 2.3. Operative Techniques

Our routine AVP and VSRR technique was described in detail previously [6]. Briefly, when the aortic root was dilated (≥45 mm), VSRR using a tube graft (Intergard; Maquet, Rastatt, Germany) and external suture annuloplasty with a CV-0 expanded polytetrafluoroethylene suture (Gore-Tex; W.L. Gore & Associates, Inc., Flagstaff, AZ, USA) were performed. In cases with a more than moderate degree of AR with a normal-size aortic root, AVP with double annuloplasty (external suture annuloplasty and sinotubular junction (STJ) remodelling using a tube graft) was performed. When external suture annuloplasty was performed, the suture was circularly passed around the outside of the root at the level of the basal ring and tied around a Hegar dilator (TK (Takanashi-Kunihara)-sizer; MA Corp., Chiba, Japan, distributed by JP Creed Corp., Tokyo, Japan) [7].

Aortic cusp configuration was evaluated carefully, and the effective height (<9 mm was defined as cusp prolapse [1]), geometric height, and free margin length (since 2018) were measured intraoperatively [8]. A glutaraldehyde-soaked autologous pericardial patch (aPP) (0.6% for 5 min) was used to fill perforation site or defects, or to perform the tricuspidization of BAV.

### 2.4. Echocardiographic Assessments

The diameter of the ventriculoaortic junction, sinus of Valsalva, and STJ were measured by preoperative transoesophageal echocardiography. Preoperative or postoperative AVA was calculated by using the continuity equation method.

Based on a previous study [5], mid-term outcomes were compared within Group-PB according to the peak PG—high-PG (subgroup-H) ≥20 mmHg and low-PG (subgroup-L) <20 mmHg. In Group-PB, the left ventricular (LV) reverse remodelling rate (%) (=[postoperative LV dimensions − preoperative LV dimensions]/[preoperative LV dimensions] × 100) was also calculated. 

### 2.5. Clinical End Points

The primary end point was to reveal the haemodynamic differences between AVP for BAV, AVP for tricuspid aortic valve, and AVR. The secondary end points were the identification of risk factors associated with a higher PG after AVP in BAV patients and freedom from AR >II recurrence and reoperation on the aortic valve within Group-PB.

### 2.6. Statistical Analysis

All continuous values are expressed as the median (interquartile range) and all categorical values are expressed as the number (percentage). The patient characteristics and operative details among the three groups were compared by analysis of variance. The Kruskal–Wallis test was used for the comparison of the continuous variables, and the χ^2^ test was used for the comparison of the frequencies between groups. 

We used propensity scoring analysis by the inverse probability of treatment weighting (IPTW) using the average treatment effect (ATE) weight method. The propensity score was calculated using logistic regression adjusted for age, sex, body surface area, LV ejection fraction (LVEF), LV diastolic diameter (LVDd), and LV systolic diameter (LVDs) in Group-PT vs. Group-R and Group-PB vs. Group-R, and age, sex, body surface area, LVEF, LVDd, LVDs, mean PG, peak PG, peak jet velocity (Vmax), and AVA in Group-PT and Group-PB. A covariate balance plot was created to examine the covariate balance before and after weighting based on propensity score.

Additionally, we explored associated factors using univariable and multivariable linear regression modelling to determine coefficients, including 95% confidence intervals (CIs) for peak PG at 7 days. The variables included in the multivariable models were determined using a stepwise method. These analyses were performed within the cases since 2018 due to missing free margin length values. The Kaplan–Meier method was used to evaluate time-dependent variables, and comparisons were performed using the log-rank test of equality. If the revision of AVP was performed during the same period of hospitalisation, freedom from reoperation and AR >II were defined according to the day of the second operation.

In post hoc subgroup analyses, no statistical hypothesis was set, and multiplicity was not accounted for, because all analyses were performed in an exploratory manner. 

All statistical tests were two-sided, and statistical significance was set at *p* < 0.05. Statistical analysis was performed with JMP 15.0 and SAS version 9.4 (SAS Institute Inc., Cary, NC, USA) and R version 4.1.2.

## 3. Results

### 3.1. Baseline Characteristics

In this analysis, patients who underwent reoperation on the aortic valve within one year (Group-PB: one patient and Group-PT: three patients) were excluded. All excluded cases developed AR after surgery, with one case in Group-PB who had aortitis and another case in Group-PT who had Loeys–Dietz syndrome. A total of 108 patients who underwent AVP, 41 patients were classified into Group-PB, and 67 patients were classified into Group-PT. There were 132 patients in Group-R. The patient characteristics are shown in Table 1. The peak/mean PG and Vmax were significantly higher in Group-PB than in Group-PT.

Operative details are also shown in Table 1. There was no significant difference in the type of AVP (AVP or VSRR) between Group-PB and Group-PT. Cusp plication, external suture annuloplasty, and STJ remodelling were performed frequently in both groups. An aPP was also used in both groups. Tricupidization was performed in two patients in Group-PB. The majority of patients in Group-R received a bioprosthetic valve (70.5%).

### 3.2. Postoperative Echocardiographic Findings

Follow-up transthoracic echocardiography (TTE) was completed at 7 days in all patients and performed at 1 year in 193 patients (79.1%). The postoperative echocardiographic findings are shown in Table 2.

At 7-day follow-up (Table 3), the results of the two-group comparison between Group-PT and -R, Group-PB and -R, and Group-PT and -PB in terms of Vmax, peak PG, and mean PG showed that the Vmax and PG were lower in Group-PT compared to in -R and -PB. Conversely, the Vmax and PG in Group-PB were higher than those in -PT and -R. Regarding AVA, the two-group comparison results for each group indicated that the AVA of Group-PT was larger than that of -R and -PB. Conversely, the AVA of Group-PB was smaller than that of -R and -PT. The findings at 1 year were similar to those at 7 days.

### 3.3. Postoperative Echocardiographic Findings Using the IPTW Method

We used the propensity scoring analysis by the IPTW method to balance the data (Figure S1).

The postoperative echocardiographic findings using the IPTW method showed that the Vmax and PG were lower in Group-PT compared to -R and -PB at 7 days (Table 3). Conversely, the Vmax and PG in Group-PB were higher than those in -PT and -R at 7 days. However, there were no significant differences in the haemodynamic parameters between Group-PB and -PT at 1 year.

### 3.4. Risk Factors for Higher Peak PG After AVP in BAV Patients

A preoperative higher Vmax, higher peak PG, smaller ventriculoaortic junction, shorter geometric height, shorter free margin length, smaller external suture annuloplasty size/body surface area (external suture annuloplasty size index), cusp plication, and use of the aPP were significantly correlated with a higher postoperative peak PG in BAV patients (Table 4). Multiple stepwise regression analysis showed that a shorter geometric height (*p* = 0.046), shorter free margin length (*p* = 0.010), and use of the aPP (*p* < 0.001) were independently correlated with a higher postoperative peak PG (postoperative peak PG = 83.307 − 1.315 × geometric height − 0.806 × free margin length − 25.290 × use of the aPP) (use of the aPP: yes = 1, no = 0). 

In Group-PB, cusp plication was performed on the fused cusp in 38 patients (92.7%), while cusp plication on the non-fused cusp was performed in 25 patients (61.0%). However, there were no significant differences in peak PG (*p* = 0.802), mean PG (*p* = 0.635), Vmax (*p* = 0.835), or AVA (*p* = 0.454), regardless of whether cusp plication was performed on the non-fused cusp or not.

### 3.5. LV Reverse Remodelling in Patients with BAV

**Figure 1:**
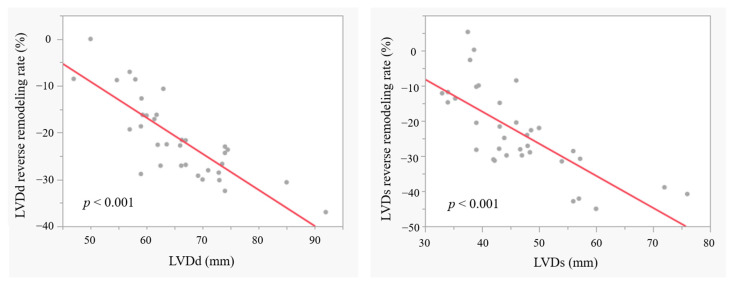
The LV reverse remodelling rates in LVDd and LVDs. LV: left ventricular; LVDd: left ventricular diastolic diameter; LVDs: left ventricular systolic diameter.

In Group-PB, the mean LV reverse remodelling rates were −22.6 (−28.0–−16.2)% (LVDd) and −24.8 (−30.8–−14.0)% (LVDs) at 1 year. The LV reverse remodelling rates in LVDd and LVDs were greater in patients with a larger preoperative LV dimension (both *p* < 0.001) (Figure 1); however, the postoperative peak/mean PG at 7-day TTE did not affect the LV reverse remodelling rate (LVDd: *p* = 0.815/0.778, LVDs: *p* = 0.993/0.772).

### 3.6. Mid-Term Outcomes in AVP for BAV

In the analysis of postoperative outcomes in Group-PB, we included a total of 42 patients. The mean follow-up duration for Group-PB was 27.5 (13.2–46.0) months. No patients died within 30 days after surgery. During the follow-up period, only one patient in Group-PB with coexisting aortitis syndrome died of acute myocardial infarction at 28.2 months after surgery. The actuarial survival rate was 95.2% at both 3 and 5 years (Figure S2a). AR >II developed in three patients during follow-up, two of whom required reoperation. The first of these patients developed AR due to aortic root destruction and multiple holes in all cusps after isolated BAV repair due to aortitis syndrome and underwent aortic root replacement at 7.1 months after surgery. This was the only patient who died, as described above. The second patient had recurrent AR again due to annular dilatation and fused cusp prolapse, despite re-AVP due to a tear in the fused cusp during the same period of hospitalisation after first AVP. This patient underwent AVR at 87.2 months after the initial surgery. The third patient developed AR due to an inadequate leaflet coaptation of the commissure at 63.0 months after the tricuspidization of very asymmetrical BAV using the aPP, and has been followed-up annually. The overall actuarial freedom from AR >II was 94.2% at both 3 and 5 years (Figure S2b) and the overall actuarial freedom from reoperation on the aortic valve was 97.2% at both 3 and 5 years (Figure S2c). Neither risk factors for AR recurrence nor reoperation could be detected.

### 3.7. Subgroup Analysis in AVP for BAV According to Postoperative Peak PG

The mid-term outcomes were compared within Group-PB according to the postoperative peak PG—subgroup-H (*n* = 20) vs. subgroup-L (*n* = 22) (Table 5). The preoperative peak/mean PG, Vmax, and AVA were not different between the two subgroups. However, the preoperative aortic root size and external suture annuloplasty size index were larger in subgroup-L. Subgroup-H showed satisfactory LV reverse remodelling in LVDd (*p* < 0.001 at 1 year) and LVDs (*p* < 0.001 at 1 year) (Table 5).

**Figure 2:**
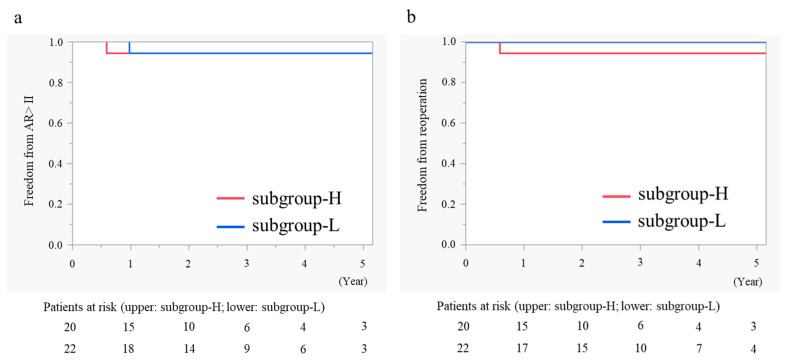
Kaplan–Meier curves showing the differences in freedom from AR >II (**a**) and freedom from reoperation on the aortic valve (**b**) between subgroup-H and subgroup-L. AR: aortic regurgitation.

The overall actuarial freedom from AR >II at 5 years was 94.4% in both subgroups (Figure 2a) and freedom from reoperation on the aortic valve at 5 years was 94.4% in subgroup-H and 100% in subgroup-L (Figure 2b); there were no differences between the groups (*p* = 0.749 and *p* = 0.317, respectively).

## 4. Discussion

This study showed that AVP for tricuspid aortic valve was more advantageous with regard to valve function compared to AVP for BAV and AVR in the early postoperative period, while AVP for BAV was associated with the risk of a higher postoperative PG compared to the other procedures. Multivariable analysis showed that a shorter geometric height, shorter free margin length, and use of the aPP were correlated with a higher postoperative PG. However, a higher postoperative PG after AVP for BAV did not affect the mid-term outcomes in this study.

### 4.1. Advantages of AVP with Regard to Valve Function

With increasing numbers of studies on AVP and its long-term outcomes [9], AVP has recently emerged as a reliable and reproducible technique. AVP has received class I recommendation in the current guidelines [10], thus providing a reasonable alternative to AVR, especially for young patients [11]. The majority of AR candidates for surgery are relatively young, and are, therefore, more likely to have complications related to the prosthetic valve if AVR is performed [12]. A recent meta-analysis showed that VSRR was associated with lower incidences of late death, thromboembolism, and bleeding events and had a similar valve durability to the Bentall operation [13]. Stent-posts supporting prosthetic valves were reported to obstruct blood flow, with a reduction in the effective orifice area by 40–70% of the total area occupied by the native valve [4]. Our results supported these findings and suggested that AVP for tricuspid aortic valve may be preferable to AVR with regard to valve function.

### 4.2. Higher PG After AVP in BAV Patients

The long-term outcomes of AVP for BAV are still controversial compared to those for tricuspid aortic valve [14]. Patlolla et al. reported that disease progression with calcification or fibrosis is the most common cause of valve failure after AVP for BAV [15]. Vohra et al. reported that leaflet calcification and BAV were risk factors for a residual higher PG, which affected valve durability [5]. Patients with BAV are more prone to a residual higher PG because they require complex repair more frequently than patients with tricuspid aortic valve [16,17,18]. Our BAV patients also required significantly more cusp plication than the tricuspid aortic valve patients, and, therefore, PG after AVP was significantly higher, although the rate of aPP use was lower in the BAV patients. Although there were no significant differences between Group-PT and Group-PB in peak/mean PG, Vmax, or AVA at 1 year in the propensity scoring analysis, these parameters may increase over time in Group-PB.

We identified short cusp geometry as a risk factor for postoperative stenosis. Schäfers et al. suggested that a geometric height of at least 16 mm in tricuspid aortic valve and 20 mm in BAV are necessary to achieve durable repair [19]. Pettersson et al. reported that shortening the free margin length (overcorrection) reduced regurgitation, but caused AVA reduction and a higher PG [16]. These results can be explained simply as BAV requires cusp plication to the fused cusp with reference to the non-fused cusp, and, therefore, a short free margin length of the non-fused cusp may lead to restriction of the motion of the fused cusp, resulting in a higher PG. To resolve this issue, the aPP may be used to extend and augment the cusp (e.g., tricuspidization). However, the use of the aPP was shown previously and in our study to be a negative prognostic factor for the long-term durability of repair [20]. These results suggest that a shorter geometric height and shorter free margin length should be managed carefully, and the use of the aPP should be avoided as much as possible.

### 4.3. Mid-Term Outcomes After AVP in BAV Patients

Postoperative residual PG should be prevented, because a higher PG negatively affects long-term outcomes, although only a few studies have discussed the impact of residual PG on clinical outcomes [5]. Vohra et al. reported that patients with a postoperative PG of ≥20 mmHg were more likely to have AR recurrence and require aortic valve reintervention during follow-up [5]. In the present study, however, PG ≥20 mmHg did not affect the mid-term outcomes and provided satisfactory LV reverse remodelling in BAV patients. Some studies have reported that postoperative LV reverse remodelling was one of the predictors of long-term outcomes, such as cardiac death or hospitalisation due to heart failure [21]. Amano et al. demonstrated that a late recurrence of LV dysfunction often occurred after AVR, and preoperative and follow-up echocardiographic parameters were predictors of the recurrence of LV dysfunction [22]. However, recent studies have shown that cardioprotective drugs play a significant role in the treatment of heart failure [23]. Mylonas et al. reported that Sodium–Glucose Cotransporter 2 Inhibitors were detected in the myocardial tissue of patients who underwent valvular surgery and were associated with myocardial remodelling and inflammation [23]. Unfortunately, our study did not account for the history of medication. The introduction and continuation of cardioprotective drugs after surgery may influence the LV reverse remodelling rate and long-term prognosis. Further studies in this area are warranted.

The inconsistency between previous studies and our study can be explained by the following reasons: Vohra et al. included both BAV and tricuspid aortic valve patients in their study. Additionally, the high-PG group (PG ≥ 20 mmHg) in their study had a significantly higher proportion of BAV patients compared to the low-PG group (PG < 20 mmHg) and more frequently underwent decalcification and shaving procedures (29.2%) than in our study. These suggest that BAV and advanced valve degeneration influenced their results. In contrast, our study focused on a younger cohort with only BAV patients and included fewer decalcification or shaving procedures (two patients in subgroup-H and two patients in subgroup-L). However, our study had a shorter follow-up period than Vohra et al.’s study. In patients with a high PG, the repair may not remain durable in the long term, potentially leading to insufficiency or the progression of valve stenosis and calcification [5]. Patlolla et al. also demonstrated that disease progression with calcification or fibrosis is the most common cause of valve failure after initial AVP for BAV, and the cumulative incidence of reoperation after initial valve repair was 11.1%, 35.9%, and 57.5% at 5 years, 10 years, and 15 years, respectively [15]. Their findings suggest that dysfunction due to valve degeneration progressively increases in the long term. Further studies with a longer follow-up are needed to validate our findings.

### 4.4. Limitations

This study had some limitations. First, the patient cohorts were not coincident in time. All cases with AR underwent AVR before 2014, when AVP had not been introduced in our facilities. In addition, there was only one surgeon for AVP (T.K.), while different surgeons performed AVR depending on the time period. Moreover, there were variations in the types of prosthetic valves used. These factors may introduce some degree of bias in the results. Second, this was a retrospective study with a small sample size. Third, this study consisted of three groups, Group-PB, Group-PT, and Group-R. BAV patients are younger and anatomically distinctive, thus, we employed propensity score analysis in the echocardiographic analysis; however, significant differences in backgrounds may introduce bias. Fourth, preoperative haemodynamic evaluations were not listed for Group-R (Table 1), because routine evaluations were not previously performed in AR patients and there were many missing haemodynamic values for Group-R. Therefore, haemodynamic variables were also excluded when adjusting between Group-R and the other groups. In addition, anatomical measurements of the aortic root (STJ, Valsalva, and ventriculoaortic junction) were collected in Group-PB, but not in Group-R. In Group-PT, we were unable to collect data from all cases. Then, in the comparison between Group-PB and -PT, we matched haemodynamic parameters, allowing for a reasonably accurate comparison. Fifth, we counted VSRR as AVP, while the type of procedure (isolated AVP or VSRR) may affect the postoperative haemodynamics. Therefore, we included the type of AVP (isolated AVP or VSRR) as a clinical variable in the analysis (Table 4). Sixth, atrial fibrillation may impact haemodynamics. While there were no cases of atrial fibrillation in Group-PB, there were seven cases (10.4%) in Group-PT. There were no data for comorbidities in Group-R. This introduces a potential source of bias. Seventh, this was a mid-term follow-up study, which should be validated in further studies with longer follow-ups.

## 5. Conclusions

AVP for tricuspid aortic valve showed favourable valve function in the early postoperative period compared to that of AVP for BAV or AVR. 

AVP for BAV is associated with the risk of a higher PG after surgery. A shorter geometric height, shorter free margin length, and use of the aPP carry risks of a higher PG. A higher PG (≥20 mmHg) after AVP for BAV did not affect the mid-term outcomes. However, further studies with longer follow-ups are needed to validate our findings.

## Figures and Tables

**Table 1:** Patient characteristics and operative details.

	AVP (*n* = 108)		AVR (*n* = 132)	*p*-Value
	Group-PB (*n* = 41)	Group-PT (*n* = 67)	Group-R (*n* = 132)	
Patient characteristics				
Age	40.0 (26.0–49.0)	57.0 (50.0–66.0)	65.5 (55.0–73.0)	<0.001
Body surface area (m^2^)	1.81 (1.72–1.91)	1.78 (1.62–1.90)	1.71 (1.56–1.85)	0.002
Male sex (%)	40 (97.6)	58 (87.9)	106 (80.3)	0.019
Preoperative echocardiography				
LVDd (mm)	65.8 (59.1–72.9)	59.4 (53.0–67.0)	65.0 (60.0–69.0)	0.002
LVDs (mm)	46.5 (39.4–52.5)	40.0 (35.6–48.3)	47.0 (41.0–52.0)	0.001
LVEF (%)	57.6 (51.0–60.2)	58.3 (52.2–62.1)	53.0 (47.0–61.0)	0.119
Peak PG (mmHg)	17.0 (11.8–23.2)	8.6 (5.1–12.7)		<0.001
Mean PG (mmHg)	9.1 (6.5–12.3)	4.4 (2.8–7.1)		0.001
Vmax (m/s)	2.2 (1.8–2.4)	1.6 (1.2–2.0)		<0.001
AVA (cm^2^)	3.4 (2.9–4.2)	3.5 (2.9–4.1)		0.804
Operations				
VSRR (remodelling) (%)	16 (39.0)	29 (43.3)		0.663
AVP (%)	25 (61.0)	38 (56.7)		
AVP details				
Cusp plication (%)	38 (92.7)	46 (68.7)		0.004
External suture annuloplasty (%)	41 (100)	59 (88.1)		0.022
External suture annuloplasty size (mm)	18 mm:1 20 mm: 5 22 mm: 19 24 mm: 16	18 mm: 4 20 mm: 21 22 mm: 29 24 mm: 4 30 mm: 1		0.002
STJ remodelling (%)	36 (87.8)	59 (88.1)		0.969
STJ remodelling size (mm)	20 mm: 1 22 mm: 4 24 mm: 18 26 mm: 13	20 mm: 1 22 mm: 12 24 mm: 38 26 mm: 7 30 mm: 1		0.064
Use of the aPP (%)	6 (14.6)	15 (22.4)		0.323
AVR operation			132	
Bioprosthetic valve (%)			93 (70.5)	
Mechanical valve (%)			39 (29.5)	
Aortic prosthetic valve size (mm)			19 mm: 5 21 mm: 9 23 mm: 34 25 mm: 53 27 mm: 31	

All continuous values are expressed as the median (interquartile range). aPP: autologous pericardial patch; AVP: aortic valvuloplasty; AVR: aortic valve replacement; LVDd: left ventricular diastolic diameter; LVDs: left ventricular systolic diameter; LVEF: left ventricular ejection fraction; Vmax: peak jet velocity; VSRR: valve-sparing aortic root replacement.

**Table 2:** Postoperative echocardiographic findings.

	Group-PB (*n* = 41)	Group-PT (*n* = 67)	Group-R (*n* = 132)
7-day TTE			
Aortic regurgitation grade (>II) (%)	0 (0)	0(0)	0 (0)
LVDd (mm)	54.0 (50.3–58.4)	49.8 (47.0–55.0)	52.0 (48.0–56.2)
LVDs (mm)	41.0 (35.0–47.3)	36.5 (32.0–43.0)	39.0 (33.8–45.0)
LVEF (%)	45.0 (35.1–58.0)	54.0 (41.0–60.0)	50.0 (37.0–59.0)
Peak PG (mmHg)	19.1 (13.2–25.3)	8.8 (6.2–12.2)	13.6 (10.7–17.9)
Mean PG (mmHg)	11.7 (7.6–15.0)	5.1 (3.4–6.4)	7.5 (6.0–10.7)
Vmax (m/s)	2.2 (1.9–2.5)	1.5 (1.4–1.9)	1.9 (1.6–2.3)
AVA (cm^2^)	1.7 (1.4–2.1)	2.6 (2.1–3.1)	1.8 (1.5–2.2)
1-year TTE			
Aortic regurgitation grade (>II) (%)	1 (2.9)	2 (3.3)	0 (0.0)
LVDd (mm)	51.0 (49.0–53.0)	48.7 (44.5–51.5)	48.0 (44.0–51.0)
LVDs (mm)	34.4 (31.0–37.6)	30.5 (28.0–34.0)	31.9 (27.3–35.0)
LVEF (%)	59.0 (56.5–64.0)	63.0 (60.3–67.7)	64.0 (58.3–69.0)
Peak PG (mmHg)	20.0 (13.0–28.9)	8.4 (6.0–12.0)	15.6 (10.5–20.2)
Mean PG (mmHg)	11.6 (7.7–15.8)	4.4 (3.0–6.1)	8.2 (5.7–11.3)
Vmax (m/s)	2.4 (1.9–2.9)	1.7 (1.4–1.8)	2.1 (1.6–2.3)
AVA (cm^2^)	2.1 (1.5–2.6)	2.6 (2.1–3.0)	2.0 (1.6–2.3)

All continuous values are expressed as the median (interquartile range). AVA: aortic valve area; LVEF: left ventricular ejection fraction; LVDd: left ventricular diastolic diameter; LVDs: left ventricular systolic diameter; PG: pressure gradient; TTE: transthoracic echocardiography; Vmax: transaortic velocity.

**Table 3:** Comparison of postoperative echocardiography findings between groups.

Evaluated Time Point	Outcome Type	Outcome	Unadjusted (*n* = 173)				IPTW (*n* = 169)			
7-day TTE	Ordered category		Odds ratio	95% CIs		*p*-value	Odds ratio	95% CIs		*p*-value
		Aortic regurgitation grade	1.44	0.691	3.004	0.331	1.278	0.776	2.105	0.335
	Continuous		Coefficient	95% CIs		*p*-value	Coefficient	95% CIs		*p*-value
		Peak PG (mmHg)	5.373	2.488	8.258	<0.001	5.467	3.016	7.918	<0.001
		Mean PG (mmHg)	3.373	1.702	5.043	<0.001	3.413	1.999	4.826	<0.001
		Vmax (m/s)	0.303	0.116	0.489	0.002	0.345	0.171	0.520	<0.001
		AVA (cm^2^)	−0.168	−0.397	0.060	0.147	−0.322	−0.534	−0.111	0.003
1-year TTE	Ordered category		Odds ratio	95% CIs		*p*-value	Odds ratio	95% CIs		*p*-value
		Aortic regurgitation grade	0.356	0.164	0.770	0.009	0.476	0.283	0.799	0.005
	Continuous		Coefficient	95% CIs		*p*-value	Coefficient	95% CIs		*p*-value
		Peak PG (mmHg)	5.402	2.146	8.658	0.001	5.647	2.833	8.462	<0.001
		Mean PG (mmHg)	3.250	1.367	5.133	<0.001	3.504	1.923	5.084	<0.001
		Vmax (m/s)	0.357	0.048	0.667	0.025	0.588	0.298	0.878	<0.001
		AVA (cm^2^)	0.335	0.036	0.633	0.028	0.105	−0.214	0.423	0.517
Evaluated time point	Outcome type	Outcome	Unadjusted (*n* = 199)				IPTW (*n* = 192)			
7-day TTE	Ordered category		Odds ratio	95% CIs		*p*-value	Odds ratio	95% CIs		*p*-value
		Aortic regurgitation grade	0.595	0.325	1.091	0.094	0.437	0.29	0.661	<0.001
	Continuous		Coefficient	95% CIs		*p*-value	Coefficient	95% CIs		*p*-value
		Peak PG (mmHg)	−4.810	−7.111	−2.509	<0.001	−4.827	−6.887	−2.768	<0.001
		Mean PG (mmHg)	−2.980	−4.261	−1.699	<0.001	−2.899	−4.014	−1.785	<0.001
		Vmax (m/s)	−0.282	−0.448	−0.116	0.001	−0.261	−0.431	−0.091	0.003
		AVA (cm^2^)	0.618	0.419	0.817	<0.001	0.453	0.268	0.638	<0.001
1-year TTE	Ordered category		Odds ratio	95% CIs		*p*-value	Odds ratio	95% CIs		*p*-value
		Aortic regurgitation grade	0.234	0.117	0.465	<0.001	0.281	0.178	0.445	<0.001
	Continuous		Coefficient	95% CIs		*p*-value	Coefficient	95% CIs		*p*-value
		Peak PG (mmHg)	−6.451	−8.671	−4.230	<0.001	−6.417	−8.476	−4.357	<0.001
		Mean PG (mmHg)	−3.603	−4.868	−2.338	<0.001	−3.636	−4.811	−2.460	<0.001
		Vmax (m/s)	−0.291	−0.569	−0.014	0.040	−0.172	−0.490	0.147	0.285
		AVA (cm^2^)	0.659	0.449	0.869	<0.001	0.636	0.434	0.837	<0.001
Evaluated time point	Outcome type	Outcome	Unadjusted *(n* = 108)				IPTW (*n* = 52)			
7-day TTE	Ordered category		Odds ratio	95% CIs		*p*-value	Odds ratio	95% CIs		*p*-value
		Aortic regurgitation grade	2.285	1.022	5.108	0.044	0.708	0.308	1.629	0.417
	Continuous		Coefficient	95% CIs		*p*-value	Coefficient	95% CIs		*p*-value
		Peak PG (mmHg)	10.184	7.004	13.363	<0.001	5.586	−0.106	11.277	0.054
		Mean PG (mmHg)	6.353	4.475	8.232	<0.001	3.827	0.663	6.992	0.019
		Vmax (m/s)	0.585	0.404	0.765	<0.001	0.337	0.028	0.647	0.033
		AVA (cm^2^)	−0.786	−1.050	−0.522	<0.001	−0.264	−0.659	0.132	0.187
1-year TTE	Ordered category		Odds ratio	95% CIs		*p*-value	Odds ratio	95% CIs		*p*-value
		Aortic regurgitation grade	1.272	0.571	2.835	0.556	0.582	0.268	1.263	0.171
	Continuous		Coefficient	95% CIs		*p*-value	Coefficient	95% CIs		*p*-value
		Peak PG (mmHg)	11.853	8.347	15.358	<0.001	3.323	−2.536	9.182	0.259
		Mean PG (mmHg)	6.853	4.829	8.876	<0.001	2.893	−0.413	6.200	0.085
		Vmax (m/s)	0.649	0.386	0.912	<0.001	0.148	−0.239	0.536	0.442
		AVA (cm^2^)	−0.324	−0.713	0.065	0.101	0.335	−0.256	0.925	0.259

AVA: aortic valve area; IPWT: inverse probability of treatment weighting; PG: pressure gradient; TTE: transthoracic echocardiography; Vmax: peak jet velocity; 95% CIs: 95%Confidence Intervals.

**Table 4:** Correlation between pre- and intraoperative valuables and postoperative pressure gradient (mmHg).

	Univariable Linear Regression				Stepwise Multivariable Linear Regression (*R*2 *=* 0.806)					
	Coefficient	95%CIs		*p* Value	Coefficient	95%CIs		*p* Value	Std Coefficient	VIF
Age	−0.073	−0.389	0.244	0.638						
Body surface area	18.693	−6.562	43.947	0.139						
Male sex	8.268	−11.876	28.413	0.403						
Ventriculoaortic junction	−1.843	−3.310	−0.376	0.016						
Sinus of Valsalva	−0.352	−0.810	0.106	0.125						
STJ	−0.289	−0.928	0.350	0.356						
LVDd	−0.352	−0.992	0.288	0.266						
LVDs	−0.362	−0.838	0.113	0.128						
LVEF	0.218	−0.465	0.900	0.515						
Peak PG	0.330	0.050	0.611	0.023						
Vmax	6.205	0.219	12.191	0.043						
AVP or VSRR	−7.218	−16.805	2.369	0.132						
Cusp plication	−14.667	−27.919	−1.414	0.032						
External suture annuloplasty size index	−3.426	−6.826	−0.027	0.048						
Geometric height	−1.805	−3.462	−0.149	0.034	−1.315	−2.605	−0.025	0.046	−0.303	1.502
Free margin length	−0.896	−1.663	−0.130	0.024	−0.806	−1.388	−0.225	0.010	−0.415	1.524
Use of the aPP	27.905	11.795	44.014	0.002	25.290	14.689	35.891	<0.001	0.586	1.024

aPP: autologous pericardial patch; AVP: aortic valvuloplasty; External suture annuloplasty size index: external suture annuloplasty size/body surface area; LVDd: left ventricular diastolic diameter; LVDs: left ventricular systolic diameter; LVEF: left ventricular ejection fraction; PG; pressure gradient; STJ: sinotubular junction; VIF: Variance inflation factor; Vmax: peak jet velocity; VSRR: valve-sparing aortic root replacement; 95%CIs: 95% Confidence Intervals.

**Table 5:** Comparison of patient characteristics, preoperative and postoperative echocardiography findings between peak PG ≥20 mmHg (subgroup-H) and peak PG <20 mmHg (subgroup-L) at 7-day TTE in patients with BAV.

	Subgroup-H (*n* = 20)	Subgroup-L (*n* = 22)	*p*-Value
Peak PG at 7-day TTE (mmHg)	25.5 (22.4–35.0)	13.3 (10.8–18.1)	
Patient characteristics			
Age (year)	41.0 (22.8–49.0)	39.0 (28.0–49.3)	0.804
Body surface area (m^2^)	1.8 (1.7–2.0)	1.8 (1.7–1.9)	0.431
Male (%)	20 (100)	21 (95.5)	0.335
Hypertension	9 (45.0)	5 (22.7)	0.126
Dyslipidaemia	2 (10.0)	0	0.129
Diabetes mellitus	0	0	
Haemodialysis	0	0	
Atrial fibrillation	0	0	
Preoperative transthoracic echocardiography			
LVDd (mm)	65.9 (59.2–73.0)	64.5 (59.0–73.0)	0.791
LVDs (mm)	46.9 (40.1–51.9)	46.0 (39.0–56.3)	0.738
LVEF (%)	58.2 (51.1–60.7)	57.5 (50.0–60.7)	0.622
Peak PG (mmHg)	17.2 (12.2–23.9)	17.5 (8.6–23.5)	0.525
Mean PG (mmHg)	9.6 (7.5–12.4)	7.5 (4.4–16.4)	0.480
Vmax (m/s)	2.2 (1.8–2.7)	2.2 (1.8–2.7)	0.571
AVA (cm^2^)	3.2 (2.2–3.9)	3.5 (3.0–4.3)	0.092
Preoperative transoesophageal echocardiography			
Ventriculoaortic junction (mm)	29.0 (28.0–30.0)	30.0 (28.0–33.0)	0.322
Sinus of Valsalva (mm)	34.0 (33.0–39.0)	38.0 (35.0–40.0)	0.019
STJ (mm)	29.0 (27.0–31.8)	30.0 (25.0–35.5)	0.200
Intraoperative cusp measurement			
Geometric height (mm)	21.5 (20.0–24.0)	24.5 (22.3–25.0)	0.017
Free margin length (mm)	38.0 (35.0–40.0)	44.5 (38.5–46.3)	0.020
Operations			
AVP: Remodelling	16: 4	10: 12	0.021
Cusp plication (%)	18 (90.0)	21 (95.5)	0.493
External suture annuloplasty size index	11.4 (10.9–13.0)	12.6 (12.4–13.4)	0.003
Use of the aPP (%)	4 (20.0)	2 (9.1)	0.313
Decalcification and shaving (%)	2 (10.0)	2 (9.1)	0.920
1-year transthoracic echocardiography			
Aortic regurgitation grade (>II) (%)	0	1 (4.5)	0.324
LVDd (mm)	52.0 ± 3.4	50.3 ± 4.1	0.174
Reverse remodelling rate (%) in LVDd	−20.6 ± 8.7	−21.9 ± 8.6	0.684
LVDs (mm)	35.1 ± 1.0	34.7 ± 1.0	0.757
Reverse remodelling rate (%) in LVDs	−24.7 ± 12.2	−23.1 ± 10.9	0.699
LVEF (%)	59.7 ± 5.8	58.7 ± 9.8	0.717
Peak PG (mmHg)	25.3 ± 11.1	17.4 ± 8.7	0.029
Mean PG (mmHg)	13.8 ± 6.5	9.7 ± 5.1	<0.001
Vmax (m/s)	2.5 ± 0.6	2.2 ± 0.5	0.094
AVA (cm^2^)	2.0 ± 0.9	2.7 ± 1.2	0.094

All continuous values are expressed as the median (interquartile range). aPP: autologous pericardial patch; AVA: aortic valve area; AVP: aortic valvuloplasty; external suture annuloplasty size index: external suture annuloplasty size/body surface area; LVEF: left ventricular ejection fraction; LVDd: left ventricular diastolic diameter; LVDs: left ventricular systolic diameter; PG: pressure gradient; STJ: sinotubular junction; Vmax: peak jet velocity.

## Data Availability

The original contributions presented in this study are included in the article/Supplementary Material. Further inquiries can be directed to the corresponding author.

## Supplementary Materials

The following supporting information can be downloaded at: https://www.mdpi.com/article/10.3390/jcm13247544/s1.
