# Supplementary material for: Haemodynamic Assessment and Outcomes of Aortic Valvuloplasty for Aortic Regurgitation in Patients with Bicuspid Aortic Valve
Source: J Clin Med. 2024 Dec 11;13(24):7544. doi: 10.3390/jcm13247544 (PMC11728290; doi:10.3390/jcm13247544)
Supplement: Supplementary file 1 [file jcm-13-07544-s001.zip › Figure S1.pdf]

Supplementary Material

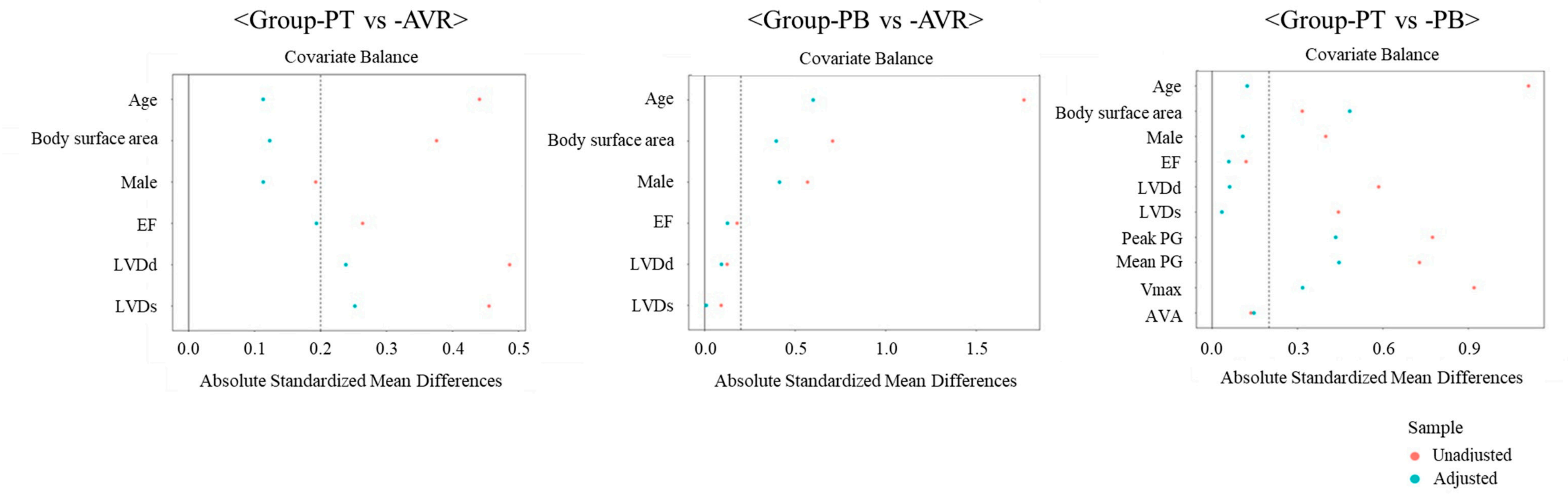

EF: Ejection fraction; LVDd: left ventricular diastolic diameter; LVDs: left ventricular systolic diameter.
