# Supplementary figures and images for: Haemodynamic Assessment and Outcomes of Aortic Valvuloplasty for Aortic Regurgitation in Patients with Bicuspid Aortic Valve
Source: J Clin Med. 2024 Dec 11;13(24):7544. doi: 10.3390/jcm13247544 (PMC11728290; doi:10.3390/jcm13247544)

Supplementary Material

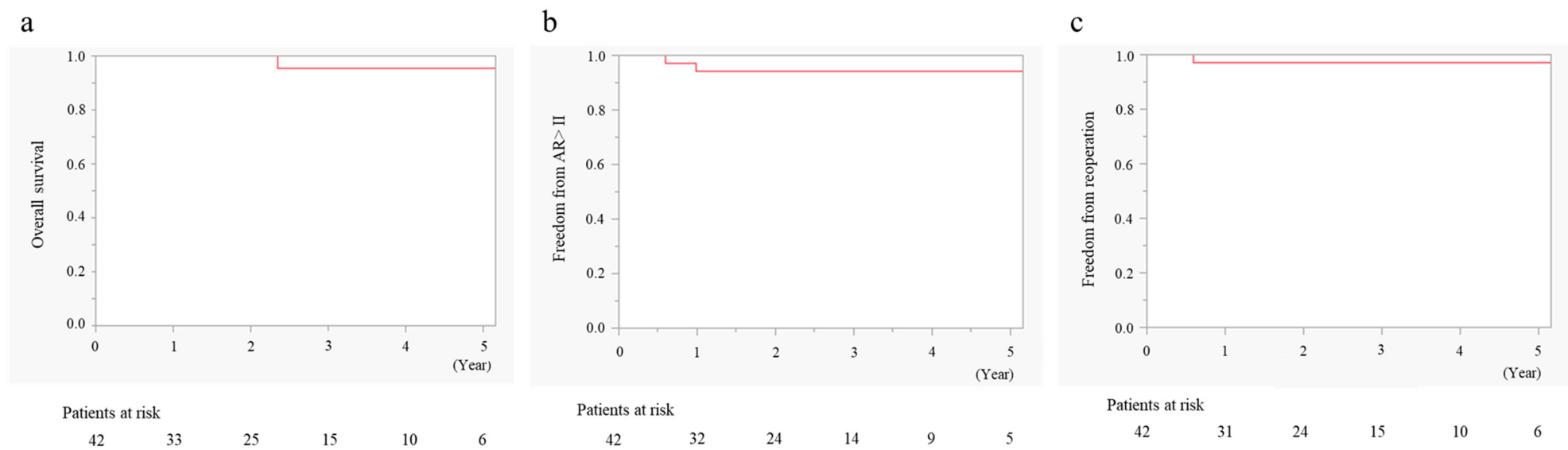

AR: aortic regurgitation; BAV: bicuspid aortic valve.

Supplement: Supplementary file 1 [file jcm-13-07544-s001.zip › Figure S2.pdf]
